# What should we consider in the case of combined Down- and 47,XY,+i(X)(q10) Klinefelter syndromes? The unique case of a male newborn and review of the literature

**DOI:** 10.1186/s12887-019-1905-9

**Published:** 2020-01-13

**Authors:** Eva Pinti, Anna Lengyel, Gyorgy Fekete, Iren Haltrich

**Affiliations:** 2nd Department of Pediatrics, Semmelweis University, Budapest, Hungary

**Keywords:** Double aneuploidy, Down syndrome, Klinefelter syndrome, Non-disjunction, Case report

## Abstract

**Background:**

Double aneuploidies - especially in combination with structural aberrations - are extremely rare among liveborns. The most frequent association is that of Down (DS) and Klinefelter syndromes (KS). We present the case of a male newborn with a unique 47,XY,+ 21[80%]/48,XY,+i(X)(q10),+ 21[20%] karyotype, hypothesize about his future phenotype, discuss the aspects of management and review the literature.

**Case presentation:**

The additional association of isochromosome Xq (i(X)(q10)) could be the result of a threefold non-disjunction event. 47,XY,+i(X)(q10) KS is not common and its symptoms differ from the classical KS phenotype. In combined DS and i(X)(q10) KS, the anticipatory phenotype is not simply the sum of the individual syndromic characteristics. This genotype is associated with higher risk for several diseases and certain conditions with more pronounced appearance: emotional and behavioral disorders; poorer mental and physical quality of life; lower muscle mass/tone/strength; connective tissue weakness; muscle hypotonia and feeding difficulties; osteopenia/−porosis with earlier beginning and faster progression; different types of congenital heart diseases; more common occurrence of hypertension; increased susceptibility to infections and female predominant autoimmune diseases; higher risk for hematological malignancies and testicular tumors.

**Conclusions:**

In multiple aneuploidies, the alterations have the potential to weaken or enhance each other, or they may not have modifying effects at all. Prenatal ultrasound signs are not obligatory symptoms of numerous chromosomal anomalies (specifically those involving supernumerary sex chromosomes), therefore combined prenatal screening has pertinence in uncomplicated pregnancies as well.

## Background

Double aneuploidies - especially in combination with structural aberrations - are extremely rare among liveborns. The most frequent co-occurrence is of Down (DS) and Klinefelter syndromes (KS) (coincidence 0.098%) [1], because they are common and relatively well tolerated chromosome abnormalities in humans [2]. Although their features are separately well described, their co-occurrence and association with i(X)(q10) structural abnormality needs a specific approach.

## Case presentation

**Fig. 1:**
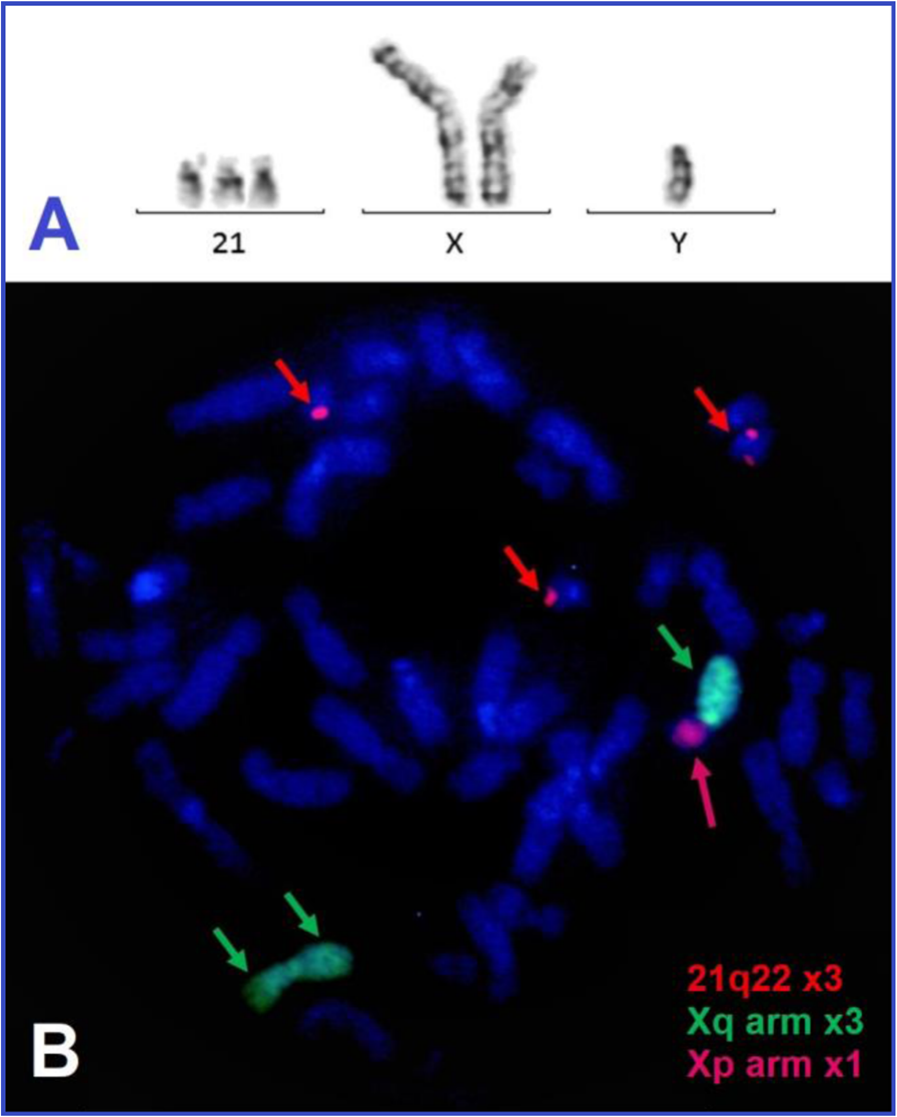
The presented patient’s genotype: 47,XY,+21[80%]/48,XY,+i(X)(q10),+21[20%]*.* **a** Partial karyogram with Giemsa-banding **b** FISH image of a metaphase cell with three 21q22 region signals (short red arrows), three Xq arm signals (short green arrows) and one Xp arm signal (long red arrow)

The 2nd child of a healthy, non-consanguineous Caucasian couple (31-year-old mother, 43-year-old father) was first referred to genetic counseling as a male newborn exclusively with signs and symptoms of DS: flat occiput, small and low-set ears, epicanthal folds, upslanting palpebral fissures, rectus diasthasis, umbilical hernia, cutis marmorata, sandal gaps on feet, moderate appetite and feeding difficulties because of generalized muscle hypotonia and a relatively large tongue. During the event-free pregnancy none of the prenatal ultrasound examinations showed any abnormalities. The child was born naturally at 39 gestational weeks with 3170 g, 54 cm, 9/10 Apgar scores and his postnatal adaptation was uneventful. Family history was negative for genetic diseases and developmental disorders and no teratogenic damage was reported. Postnatal analysis of his 15 Giemsa-stained metaphases from standard 72-h peripheral blood lymphocyte cultures led to the 47,XY,+ 21[80%]/48,XY,+i(X)(q10),+ 21[20%] genotype. The proportion of mosaicism was confirmed to be 47,XY,+ 21[80%]/48,XY,+i(X)(q10),+ 21[20%] by fluorescent in situ hybridization (FISH) of 200 interphase cells with 21q22 region (Kreatech, Amsterdam, Netherlands), Xp and Xq (Cytocell, United Kingdom) specific probes (Fig. 1). The 2-month-old child was re-examined when the genetic test results were reported. The features of DS continued to dominate his phenotype, but due to his young age we could not see any KS traits.

## Discussion and conclusions

### Incidence, etiology

Approximately 15% of clinically proven pregnancies end with spontaneous miscarriages, half of them (7.5%) arise from chromosome abnormalities. In the case of stillbirths this rate is 6–13% [3]. In spontaneous abortions the detection rate of double and multiple aneuploidies is 4.6 and 0.4%, respectively. Double aneuploidies are mostly composed (in descending frequency-order) of chromosome 21, 16, X, 22, 18, 13 and 15. The most frequent combinations are -X/+ 21 (8.5%), + 21/+ 22 (4.4%), + 16/+ 21 (4.4%), + 7/+ 16 (4.4%) in double, and + X/+ 5/+ 8, + 8/+ 20/+ 22, + 16/+ 20/+ 22, + 14/+ 21/+ 22, −X/+ 21/+ 21, −X/+ 7/+ 21 in multiple aneuploidies. These results suggest that the excess of chromosomes (with the exception of X) is less tolerated than their deficiency. The incidence of these abnormalities is significantly higher (59.2%) in maternal age group 40–44 years [4], where the higher risk of chromosome aberrations - due to the increased age (≥35 years) - combines with greater willingness to bear children in more developed countries.

The incidences of various chromosome aneuploidies differ between livebirths and spontaneous abortions or stillbirths. While autosomal trisomies in combination with X monosomy are more common in the latter group, the association of DS and KS are more dominant in the former (incidence 4–9:100000 male newborns) [2]. Co-occurrence of DS and KS is higher (9.8 × 10^− 2^) than expected (1.298 × 10^− 6^) [1] because of their similar origin: increased predisposition for non-disjunction events [5]. This hypothesis is supported by the theory that the imbalance of one chromosome increases the risk for further aberrations [6, 7]. The simultaneous appearance of our patient’s three chromosomal aberrations is therefore the result of a threefold non-disjunction event.

Cumulative cell division defects may be explainable by the presence of different epigenetic and other risk factors. The risk is higher in instances of increased maternal (≥35 years) or paternal (≥45 years) age [8], cousin marriage, known genetic disorders in the family, pathological fetal ultrasound signs, increased maternal serum marker levels, quantitative abnormalities of amniotic fluid, certain medicaments, infections, chemicals or ionizing radiation during the pregnancy.

88% of DS cases arise from the 2nd maternal and the 1st grandmaternal meiotic cell division, 8% have paternal origin and 4% occur after fertilization [9, 10]. Meiotic non-disjunction events causing KS are inherited maternally in 50–56% of cases (36% occur in 1st and 20% in 2nd meiosis), and paternally in 44–50% (100% 1st meiosis) [11, 12]. Isochromosome Xq originates presumably during the 2nd maternal meiosis from a separation error of the centromere or the sister chromatids [12].

In the presented newborn the excess of chromosome 21, as it is non-mosaic, could have originated from either a paternal or maternal germ cell, or from an early postzygotic cell division defect. In contrast, the i(X)(q10) is not present in all the lymphocytes of the patient. Some authors suggest that 47,XY,+i(X)(q10) could be maternally inherited [12], but on the basis of mosaicism a postzygotic, mitotic defect is more likely in the present case. A further explanation could be that the maternally inherited extra chromosome had been lost during somatic cell divisions as the result of a negative selection effect.

### Genotype-phenotype correlation (Table 1)

#### Down syndrome

The immune system’s dysregulation and its consequences, as well as the higher risk for infections and some types of leukemias are characteristic for DS [13, 14]. A further, scarcely understood aspect is that patients with DS have decreased risk for certain solid tumors, a notable exception being testicular cancer [15]. This relative protection could be explained by higher dosages of putative tumor suppressor loci on chromosome 21 [16, 17].

**Table 1:** Summary of the most important phenotypic aspects of the discussed chromosomal abnormalities

DS	KS	i(X)(q10) KS	Predicted phenotype	Patient’s signs
Prenatal signs				
↑↑: increased maternal serum marker levels (PAPP-A, uE3, total & free β-hCG); ultrasound signs (hypoplastic nasal bone, >3mm nuchal translucency, abnormal ductus venosus flow, oligo-/polyhydramnios, 5th finger brachymesophalangy, ventriculomegaly, short femur & humerus)	[SGA: increases with number of extra X chromosomes]	[− SGA]	↑↑: increased maternal serum marker levels (PAPP-A, uE3, total & free β-hCG); ultrasound signs (hypoplastic nasal bone, >3mm nuchal translucency, abnormal ductus venosus flow, oligo-/polyhydramnios, 5th finger brachymesophalangy, ventriculomegaly, short femur & humerus) [SGA]	no signs
Nervous sys.				
↑↑: ID/DD; epilepsy	↑: moderate ID: increases with number of extra X chromosomes; mild language problems; deficits in executive functions; smaller total brain/gray & white matter volume	[mild language problems?; deficits in executive functions?; smaller total brain/gray & white matter volume?]	↑↑: ID/DD; epilepsy; mild language problems?; deficits in executive functions?; smaller total brain/gray & white matter volume?	no signs
Mental & socioeconomic status				
↑↑: autism spectrum disorder; good mood; Alzheimer’s disease; poorer mental & physical quality of life	↑↑: anxiety; depression; behavioral disorders; poorer mental & physical quality of life	↑↑: anxiety; depression; behavioral disorders; poorer mental & physical quality of life	↑↑↑↑: autism spectrum disorder; Alzheimer’s disease; anxiety, emotional & behavioral disorders; poorer mental & physical quality of life	no signs
Musculosceletal & connective tissue sys.				
↑↑: connective tissue weakness (rectus diasthasis, inguinal/umbilical hernia); joint hypermobility; muscle hypotonia	↑↑: low muscle mass/strength/tone; early & more pronounced osteopenia/-porosis	↑↑: low muscle mass/strength/tone; early & more pronounced osteopenia/-porosis	↑↑↑: connective tissue weakness (rectus diasthasis, inguinal/umbilical hernia); joint hypermobility; muscle hypotonia, low muscle mass/strength/tone; early & more pronounced osteopenia/-porosis	rectus diasthasis, umbilical hernia, muscle hypotonia
Hearing impairment				
↑↑: sensorineural/ conductive (frequent middle ear inflammation)	[not typical]	[not typical]	↑↑: sensorineural/ conductive (frequent middle ear inflammation)	no signs
Ophthalmic anomalies				
↑↑: Brushfield’s macules, cataract, glaucoma, keratoconus, strabismus, refractive errors	[not typical]	[not typical]	↑↑: Brushfield’s macules, cataract, glaucoma, keratoconus, strabismus, refractive errors	no signs
Cardiovascular sys.				
↑↑: AVSD; VSD; Fallot tetralogy; mitral valve problems	↑: hypertension; higher risk for DVT & PE; short QTc	↑: hypertension; higher risk for DVT & PE	↑↑↑: AVSD; VSD; Fallot tetralogy; mitral valve problems ↑: hypertension; higher risk for DVT & PE	no signs
Immune sys.				
↑↑: increased suscept. to infections	↑↑: increased suscept. to autoimmune diseases (SM, RA, Hashimoto’s disease, Sjögren’s syndrome, SLE, T1DM)	↑↑↑: increased suscept. to autoimmune diseases (SM, RA, Hashimoto’s disease, Sjögren’s syndrome, SLE,T1DM)	↑↑↑↑: increased suscept. to infections & autoimmune diseases	no signs
Endocrine sys.				
↑↑: congenital primary hypothyroidism; T1DM/T2DM	↑↑: hypergonadotropic hypogonadism; metabolic syndrome (T2DM, hypertension, dyslipidemia)	↑↑: hypergonadotropic hypogonadism; metabolic syndrome	↑↑: congenital primary hypothyroidism; T1DM/T2DM; hypergonadotropic hypogonadism; metabolic syndrome	no signs
Gastrointestinal sys.				
↑↑: constipation, decreased appetite, Hirschprung’s disease, pyloric stenosis, duodenal atresia, Meckel’s diverticulum, anal atresia, celiac disease, gastroesophageal reflux disease	[not typical]	[not typical]	↑↑: decreased appetite, constipation, Hirschprung’s disease, pyloric stenosis, duodenal atresia, Meckel’s diverticulum, anal atresia, celiac disease, gastroesophageal reflux disease	moderate appetite
Oropharyngeal abnormalities				
↑: necrotizing ulcerative gingivitis, periodontitis, early tooth loss, malocclusion, late teething, small teeth, dental enamel hypocalcification, cheilognathopalatoschisis	[not typical]	[not typical]	↑: necrotizing ulcerative gingivitis, periodontitis, early tooth loss, malocclusion, late teething, small teeth, dental enamel hypocalcification, cheilognathopalatoschisis	no signs
Fertility & sexual problems				
↑↑: decreased fertility/infertility (in male: oligo/azoospermia & in female: early menopause, decreased sexual activity)	↑↑: decreased fertility/infertility (oligo-/azoospermia, decreased sexual activity)	↑↑: decreased fertility/infertility (oligo-/azoospermia, decreased sexual activity)	↑↑↑↑: decreased fertility/infertility (oligo-/azoospermia, decreased sexual activity)	no signs
Tumor predisposition				
↑↑: increased risk for: testicular germ cell tumors, ALL, AML, AMKL	↑: risk for: extragonadal germ cell tumors & breast cancer	↑: risk for: extragonadal germ cell tumors & breast cancer	↑↑↑↑: increased risk for: extragonadal germ cell & testicular tumors, ALL, ML-DS	no signs
↓: risk for certain solid tumors: breast, lung, prostate			↓: risk for lung & prostate cancer	no signs

Table legend: square brackets []: rare/non-characteristic features, ↓: decreased risk, ↑: slightly increased risk/incidence, ↑↑: moderate increased risk/incidence, ↑↑↑: expressed risk/incidence, ↑↑↑↑: very high risk/incidence, *sy.* syndrome, *sys.* system, *PAPP-A* pregnancy-associated plasma protein A, *uE3* unconjugated oestriol, *β-hCG* beta human chorionic gonadotropin, *SGA* small for gestational age, *ID* intellectual disability, *DD* developmental delay, ? unclarified significance, *AVSD* atrioventricular septal defect, *VSD* ventricular septal defect, *DVT* deep vein thrombosis, *PE* pulmonary embolism, *QTc* corrected QT interval, *SM* sclerosis multiplex, *RA* rheumatoid arthritis, *SLE* systemic lupus erythematosus, *T1DM* type 1 diabetes mellitus, *T2DM* type 2 diabetes mellitus, *ALL* acute lymphoid leukemia, *AML* acute myeloid leukemia, *AMKL* acute megakaryoblastic leukemia, *ML-DS* myeloid leukemia of Down syndrome

Children with DS have a 20-fold greater risk for acute lymphoid leukemia (ALL) and a 500-fold higher risk for myeloid leukemia (ML) compared to children without DS. Overall survival (OS) in DS-ALL (70%) is below the group of patients without DS (OS: 89%). The increased rate of treatment-related toxicity and infectious deaths in DS-ALL can be explained by the patients’ altered metabolic profile and immunodeficient state. The long-distance outcomes are further worsened by the high risk for relapse even after hematopoietic stem cell transplantation. Minimal residual disease response can differentiate between low and high relapse risk DS-ALL patients, and indicate the necessary and safe intensity of treatment [18].

5–10% of DS newborns have manifest, and 10–31% have silent transient myeloproliferative disorder (TMD). TMD is a preleukemia characterised by *GATA Binding Protein 1* (*GATA1*) gene mutations in 30% of DS newborns. 25% of TMD in DS (DS-TMD) undergo spontaneous regression (in the first 3 months of life), while the remainder will go on to develop myeloid leukemia of DS (ML-DS). Most cases of DS-TMD are diagnosed 3–7 days after birth, but almost all of them present signs of TMD by age 2 months. 20–30% of TMD (meaning 0.5–2% of all patients with DS) will go on to develop ML-DS (within the first 2–4 years of life, approximately 1.2–1.5 years from the onset of TMD) or, less commonly, ALL. ML-DS involves two entities: myelodysplastic syndrome (MDS) and acute megakaryoblastic leukemia (AMKL). MDS often precedes AMKL [18].

There is no standard for screening *GATA1* mutations in patients with DS, but international guidelines recommend at least one full blood count (FBC) for newborns. Patients who have documented DS-TMD require regular monitoring every 3 months until 4 years of age with FBC and physical examination, due to their higher risk for developing ML-DS. ML-DS patients have significantly better disease-free survival (DFS: 88–89%) compared to children without DS (DFS: 42%), because ML-DS shows better response to less intense therapy. The better chemosensitivity of AMKL could be explained by alteration of cytarabine metabolism. Patients with DS – who develop AMKL – have better 5-years event-free survival (5yEFS: 81%) under the age of 4 years than older children (5yEFS: 33%). Children over 4 years of age with DS and AMKL are more likely to have cytogenetic aberrations similar to sporadic AML, therefore older children with AMKL need more intense therapy [18].

#### Klinefelter syndrome

47,XY,+i(X)(q10) is very rare among patients with KS. Including our patient, 23 cases have been reported so far. Although the KS encompasses a wide spectrum of features, the classical phenotype includes consequences of hypergonadotropic hypogonadism. Height can exceed the average, presumably because of delayed epiphyseal plate closure and the extra copy of *short stature homeobox* (*SHOX)* gene on Xp [19–23].

Several studies have dealt with the distinct phenotypic effect of the supernumerary X chromosome in KS. It has emerged that the X inactivation pattern of KS patients is similar to that of healthy women, which extends not only to the extra X chromosome(s), but also to the autosomes. Protection against inactivation is ensured by the expression of *X inactive specific transcript (XIST)* gene on the long arm of the active X. This mechanism regulates X-chromosomal gene dosages in relation to the number of autosomes, therefore in cases of tri-, tetra- or higher polyploidy, there are more than one active X chromosomes [24]. Therefore, the KS phenotype is influenced by more than simply the increased dosages of non-inactivated X-chromosomal genes [7, 23]. In KS patients this may lead to the increased incidence of female predominant autoimmune disorders [25].

Skakkebæk et al. analysed the genome-wide transcriptome and methylome of peripheral blood leukocytes in KS patients, male and female controls and detected a unique, KS-specific genetic and epigenetic landscape. They identified candidate genes (coding and non-coding) and regions responsible for the diverse KS features and comorbidities [23].

#### I(X)(q10) Klinefelter syndrome

In 47,XY,+i(X)(q10) some traits, but not all, could easily be originated from the altered dosages of the X chromosomal arms. The excess of Xq is responsible for most of the typical KS symptoms, and the relative lack of Xp for normal height and intellect [7, 20].

The frequency of various autoimmune diseases distributes differently between the sexes and different types of chromosome abnormalities, which illustrates the dosage-dependent effect of X-chromosomal genes. Approximately 15% of them remain active (30% of the Xp and < 3% of the Xq genes) [26]. More precisely, 5% of the genes escape completely from inactivation and 10% show variable cell-type specific patterns [7]. Decreased expression of *Forkhead Box P3* (*FOXP3*) gene on Xp11.23, which encodes a transcription factor responsible for regulatory T-cell differentiation, leads to insufficient inhibition of autoreactive T-cells. Deletion of *FOXP3* causes the IPEX syndrome, which is characterized by immune-dysregulation, polyendocrinopathy, enteropathy and X-linked inheritance. Further anti- *(Interleukin 10 (IL10), Transforming Growth Factor Beta 2 (TGFβ2))* and pro-inflammatory *(Interleukin 6 (IL6), Transforming Growth Factor Beta 1 (TGFβ1))* cytokine genes are located on the X chromosome, altered dosages of which influence the immune system’s regulation in the opposite way. The decreased dosages of anti-inflammatory cytokine genes on Xp and the increased copies of pro-inflammatory cytokine genes on Xq lead to higher risk for autoimmune diseases. This data is supported by the fact that some disorders (e.g. Hashimoto’s disease) occur more commonly among patients with 45,X,i(X)(q10) Turner syndrome [27]. A similar relationship between the altered cytokine gene dosages and higher autoimmune disease risk may exist in KS with i(X)(q10) structural abnormality.

It is not yet known how inactivation is affected in structural X abnormalities, notably in the 47,XY,+i(X)(q10) genotype. Considering the results of Skakkebæk et al., the expression of candidate Xp genes presumably involved in the KS phenotype may not be altered in the case of i(X)(q10) KS, therefore those features should be lacking. One of those genes is *eukaryotic translation initiation factor 2 subunit gamma* (*EIF2S3*)*,* which is associated with diabetes, cognitive dysfunction and altered brain volumes, hypogonadism and azoospermia in typical KS [23]. If the expression of *EIF2S3* is not altered in i(X)(q10) KS, the aforementioned symptoms should be not present. This hypothesis is supported by the fact that as of yet all reported i(X)(q10) KS patients have normal intellect. On the other hand, these patients are affected by hypogonadism and azoospermia, which further complicates the proper understanding of genotype-phenotype correlations.

#### Double 48,XXY,+ 21 trisomy

Since the first description of double 48,XXY,+ 21 trisomy [28], 82 postnatal and 17 prenatal cases have been reported [29]. Interestingly, in 1963 Wright and his colleagues reported two independent sibling duos with single aneuploidies. There was one child with KS and one with DS in each family [30], which are further examples of the common combination of these aneuploidies and the increased predisposition for non-disjunction events. Prenatal hints of similar cases could be the ultrasound signs of DS and the increased maternal serum marker levels. Keeping in mind that ultrasound signs are not obligatory, simultaneous screening of biomarker levels is essential. Prenatal ultrasound examinations of the presented patient did not show any pathological alterations. Since the mother was under 35 years of age, no serum biomarker testing was conducted. Our patient’s case supports the relevance of combined prenatal testing in uncomplicated pregnancies.

In cases of combined DS and KS the incidence of congenital heart disease could be higher compared to the isolated forms of these aneuploidies. In 12 previously described 48,XXY,+ 21 cases atrial and atrioventricular septal defects were the most frequent abnormalities [31].

In addition, the spectrum of different mental problems could be wider, and their risk could be higher in this group of patients.

The phenotype of 48,XXY,+ 21 double aneuploidy is characterized by classical DS features in children, who then develop additional KS symptoms from the age of puberty. If we experience the following symptoms, we should suspect the presence of KS beside DS: male infant with developmental abnormalities of the genitalia (hypospadias, micropenis, cryptorchidism), delay or absence of puberty, signs of hypergonadotropic hypogonadism (absence of voice cracking, female secondary sex characteristics, gynecomastia, < 4 ml testicle volume), oligo−/azoospermia, tall stature (>97th percentile).

#### Combined Down and i(X)(q10) Klinefelter syndrome

The combined DS and i(X)(q10) KS is associated with higher risk for several diseases and certain conditions with more pronounced appearance: emotional and behavioral disorders; poorer mental and physical quality of life; lower muscle mass/tone/strength; connective tissue weakness; muscle hypotonia and feeding difficulties; osteopenia/−porosis with earlier beginning and faster progression; different types of congenital heart diseases; more common occurrence of hypertension; increased susceptibility to infections and female predominant autoimmune diseases; higher risk for hematological malignancies and testicular tumors.

In multiple aneuploidies and compound chromosomal abnormalities the anticipatory phenotype is not simply the sum of the individual syndromic characteristics. The alterations have the potential to weaken or enhance each other, or they may not have modifying effects at all. Prenatal ultrasound signs are not obligatory symptoms of numerous chromosomal anomalies (specifically those involving supernumerary sex chromosomes), therefore combined prenatal screening has pertinence in uncomplicated pregnancies as well. This might also suggest that only chromosomal anomalies that usually lead to pronounced anatomical malformations (e.g. 21/13/18 trisomies) may be detected during the 18- to 22-week fetal anatomical ultrasound. In cases of i(X)(q10) KS long-term follow-up, transcriptome and methylome analyses are needed to clarify the role of the X-chromosomal regions in KS traits, the regulation of the immune system and autoimmune diseases.

## Data Availability

Not applicable.

## Abbreviations

5yEFS: 5-years event-free survival
ALL: Acute lymphoid leukemia
AMKL: Acute megakaryoblastic leukemia
AML: Acute myeloid leukemia
AVSD: Atrioventricular septal defect
DD: Developmental delay
DFS: Disease-free survival
DS: Down syndrome
DS-ALL: Acute lymphoid leukemia in Down syndrome
DS-TMD: Transient myeloproliferative disorder in Down syndrome
DVT: Deep vein thrombosis
*EIF2S3*: *Eukaryotic Translation Initiation Factor 2 Subunit Gamma* gene
FBC: Full blood count
FISH: Fluorescent in situ hybridization
*FOXP3*: *Forkhead Box P3* gene
*GATA1*: *GATA Binding Protein 1* gene
i(X)(q10): Isochromosome of the X-chromosomal long arm
ID: Intellectual dysability
*IL10*: *Interleukin 10* gene
*IL6*: *Interleukin 6* gene
IPEX syndrome: Immune-dysregulation, Polyendrocrinopathy, Enteropathy, X-linked inheritance syndrome
KS: Klinefelter syndrome
MDS: Myelodysplastic syndrome
ML: Myeloid leukemia
ML-DS: Myeloid leukemia in Down syndrome
OS: Overall survival
p: Short arm of a chromosome
PAPP-A: Pregnancy-associated plasma protein A
PE: Pulmonary embolism
q: Long arm of a chromosome
QTc: Corrected QT interval
RA: Rheumatoid arthritis
SGA: Small for gestational age
*SHOX*: *Short stature homeobox* gene
SLE: Systemic lupus erythematosus
SM: Sclerosis multiplex
sy.: Syndrome
sys.: System
T1DM: Type 1 diabetes mellitus
T2DM: Type 2 diabetes mellitus
*TGFβ1*: *Transforming Growth Factor Beta 1* gene
*TGFβ2*: *Transforming Growth Factor Beta 2* gene
TMD: Transient myeloproliferative disorder
uE3: Unconjugated oestriol
VSD: Ventricular septal defect
X: X chromosome
*XIST*: *X inactive specific transcript* gene
Y: Y chromosome
β-hCG: Beta human chorionic gonadotropin
